# Preface

**Published:** 2007

We continue the Academia-Industry Symposium with this issue.

**Jerome P. Kassirer,** Distinguished Professor at Tufts University School of Medicine, Editor-in-Chief Emeritus, *New England Journal of Medicine,* writes an editorial: ‘Extent And Implications Of The Academia-Industry Connection’ (p1-6).

**Joel Lexchin,** Professor in the School of Health Policy and Management at York University, writes another editorial: ‘Of Money And Trust In Biomedical Care’ (p7–10).

*MSM* Editors, **Ajai R. Singh** and **Shakuntala A. Singh** write the third editorial: ‘Academia, Journal Publishing and the Bio-Medical Industry’ (p11–14).

**Martin B Van der Weyden,** Editor, *Medical Journal of Australia*, writes for *The Looking Glass*: ‘The ICMJE and URM: Providing Independent Advice for the Conduct of Biomedical Research and Publication’ (p15–24).

We continue with the theme of the 2006 monograph: ‘What Medicine Means To Me’ in this issue of MSM with some important contributions.

**Lynne Layton,** Editor, *Psychoanalysis, Culture and Society* and Assistant Clinical Professor of Psychology, Harvard Medical School writes on, ‘What Psychoanalysis, Culture And Society Mean To Me’ (p146–157).

**Alfredo Pereira Jr,** currently Adjunct Professor at the State University São Paulo Júlio de Mesquita Filho and earlier, Post-Doctoral Fellow in Sciences of the Brain and Cognition at the Massachusetts Institute of Technology (1996–98), writes on, ‘What the cognitive neurosciences mean to me’ (p158–168).

**Elizabeth Wager,** freelance medical writer, editor and trainer based in Princes Risborough, England and member of the ethics committees for the BMJ and WAME (World Association of Medical Editors) and a council member of COPE (the Committee On Publication Ethics), writes on, ‘What medical writing means to me’ (p169–178).

**Helen Herrman,** Director, World Health Organisation Collaborating Center for Research and Training in Mental Health, University of Melbourne and Secretary for Publications, World Psychiatric Association, writes on, ‘What Psychiatry means to me’ (p179–187).

‘Recollections of a Journey Through a Psychotic Episode: Or, Mental Illness and Creativity,’ in *Reflections* is a scientist’s **Anonymous** contribution for obvious reasons (p188–196).

**Madhukar S. Bandisode,** earlier Chief, Intermediate Medicine and Medical Director of Extended Care Services from 1979 to 2000 (now retired) and in clinical practice, responds to questions raised in, ‘Where is Medical Practice in India Heading?’ and, ‘Turning Points in my Medical Career’, both articles in the 2006 *MSM* by Sunil K. Pandya (p197–204).

**Roy, Vance, Col. Goel, Nicole, Madhukar, Kumar, Morten, Sadhu, Shakuntala, Ajai,** members of the mensanamonographs e-group, discuss the 2006 editorial of *MSM*, ‘To Cure Sometimes, To Comfort Always, To hurt The Least, To Harm Never’ (p205–227).

**Cortney Davis,** nurse practitioner in women's health, author of three poetry collections, most recently “Leopold's Maneuvers,” winner of the Prairie Schooner Poetry Prize, writes two poems for MSM Poems: ‘Doctor At Work, Late Evening’ (p228) and ‘Taking Care of Time’ (p229).

**Ajit V. Bhide,** Head, Departments of psychiatry and Family Medicine at St. Martha's Hospital, Bangalore, India, writes an obituary, ‘Ravinder Lal Kapur, M.D. (1938–2006)’ (p231–236). The Editor also writes on Dr Kapur (pviii–ix).

This issue of MSM is dedicated to the illustrious memory of Dr. R.L. Kapur (pvii).

The Theme MSM is entitled: ‘Guidelines, Editors, Pharma And The Biological Paradigm shift’ and is authored by the **Editors of *MSM*** (p25–145). It is divided into ten chapters, complete with Musings (p25–26), an abstract (p27–30), ten chapters (p31–127), concluding remarks (p128–133), questions that this monograph raises (p134–135), and monograph references list (p136–145).

